# Sand fly behavior: much more than weak-flying

**DOI:** 10.1590/0074-02760210230

**Published:** 2021-11-19

**Authors:** Gabriel Barbosa Tonelli, Camila Binder, Carina Margonari, José Dilermando Andrade

**Affiliations:** 1 Fundação Oswaldo Cruz-Fiocruz, Instituto René Rachou, Grupo de Estudos em Leishmanioses, Belo Horizonte, MG, Brasil

**Keywords:** dispersion, flight capacity, leishmaniasis, sand flies, vectors

## Abstract

**BACKGROUND:**

Leishmaniases are diseases transmitted by some species of sand flies and are widely distributed throughout the tropical regions of the planet. Despite the low mobility of these vectors, the geographical distributions of some species are quite extensive, which hinders control and surveillance measures in endemic areas.

**OBJECTIVES:**

The present study investigated the flying capacity of sand flies.

**METHODS:**

Four Hoover Penido (HP)-type light traps were positioned in the centre of the Velhas’ River, about 80 metres equidistant from each other. We also realised capture/release/recapture attempts to assess possible capacity of phlebotomine fly uninterrupted up to 150 metres. Captured sand flies from one side of the river were marked using fluorescent powder (Luminous Paint kit, Bioquip^®^) and released on the other side, approximately 150 m distant. Recapture attempts were made on river’s bank up to 30 days post-release.

**FINDINGS:**

Six sand flies of the species *Nyssomyia neivai* (n = 4), *Ny. intermedia* (n = 1) and *Evandromyia lenti* (n = 1) were captured in the centre of the river. There were no recaptures of the 1,450 marked-and-released sand flies.

**MAIN CONCLUSIONS:**

The results obtained disagree with data found in the literature regarding the flight capacity of sand fly vectors of leishmaniasis.

Phlebotomine insects are vectors of viruses, bacteria and protozoa, with the protozoan genus *Leishmania* being the main pathogen carried by this group of insects. Sand flies are most abundant in the Neotropical Region, where they have greater species richness and densities that fluctuate according to climatic season,^1^ with approximately 40 species being involved in the transmission of the *Leishmania* parasite.^2^ ^,^ ^3^

Ecologically, sand flies are known to be poor flyers,^4^ traveling by jumping flights.^5^ Poché et al.^6^ demonstrated that *Phlebotomus argentipes* disperse among palm tree canopies, suggesting that some species of sand flies may use treetops as shelter and disperse through these microhabitats. Despite their limited mobility, several species of sand flies have broad geographical distributions, including *Lutzomyia longipalpis*, the main species involved in the epidemiology of visceral leishmaniasis.^7^ ^,^ ^8^ The broad distributions of sand flies, combined with different aspects of leishmaniasis epidemiology, has hampered control and surveillance measures in endemic areas, resulting in significant increases in cases of this disease.

Sand flies are known to disperse in urban areas, up to a few kilometres when looking for food and a few metres when not looking for food,^9^ yet there remains a knowledge gap regarding their ecology as it relates to their dispersion and flight capacity.^10^ Areas with natural geographical barriers that hinder the dispersion of sand flies, such as rivers, would be ideal places to carrying out these types of studies because, using appropriate methodologies, they would allow assessing and measuring the flight capacity of these insects.

Although sand flies are known to make short jumping-flights, we believe, based on the broad distributions of these insects throughout the world, that these dipterans can perform longer, uninterrupted flights than reported, wind aided or not. Therefore, we tested the null hypothesis (H0) that sand flies jump-fly for a few metres a time, as described in the literature; with the alternative hypothesis (H1) being that sand flies can fly continually over longer distances (Fig. 1).

**Fig. 1:**
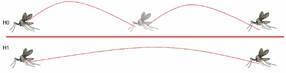
hypotheses to be tested regarding the flying ability of sand flies. Null hypothesis (H0): sand flies fly by making short jump-flights. Alternative hypothesis (H1): sand flies can make continuous long flights.

Testing the flight capacity of sand flies will contribute to knowledge about their ecology and distribution, allowing inferences to be made about the distribution of leishmaniasis and improvements to vector control actions.

## MATERIALS AND METHODS

**Fig. 2:**
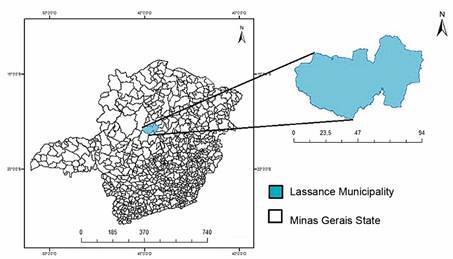
location of the municipality of Lassance in the State of Minas Gerais, Brazil.

*Study area* - The study took place on the banks of the Velhas’ River in the municipality of Lassance, located in the Northern Region of the State of Minas Gerais, Brazil (17**º** 53’ 13” S, 44**º** 34’ 40” W) (Fig. 2). The municipality encompasses 3.214 km² and has 6.554 human inhabitants.

The area of the Velhas’ River was chosen because it acts as a possible geographical barrier to sand fly dispersion and, thus, allows testing the proposed hypotheses. The river has several long and straight stretches in its course. Its greatest depth less than 15 metres and the distance between its banks in the study area reaches 180 m.

**Fig. 3:**
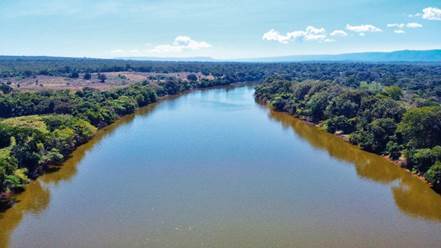
stretch of Velhas’ River selected for the distribution of automatic Hoover Penido (HP) light traps for attempted flight interception.

*Flight capacity* - The stretch of Velhas’ River selected to attempt to capture sand flies by flight intervention is more than 500 metres in length, with minimal curvature in its course, and 180 metres wide, allowing for the distribution of traps in a homogeneous way (Fig. 3).

**Fig. 4:**
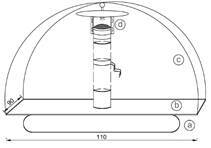
support developed to capture flying sand flies in an aquatic environment: (a) air chamber; (b) wooden plate (110 cm x 90 cm); (c) trap support wire; and (d) Hoover Penido (HP) light trap.

A support was developed to float traps in the aquatic environment of the river (Fig. 4). The supports were anchored with a rope tied to a heavy object (e.g., solid iron strips). Three collections were carried out, one in each of the months of May 2017, January 2018 and July 2018, with a sampling effort of three consecutive days per collection, totalising 72 h of sampling effort per month of collection.

**Fig. 5:**
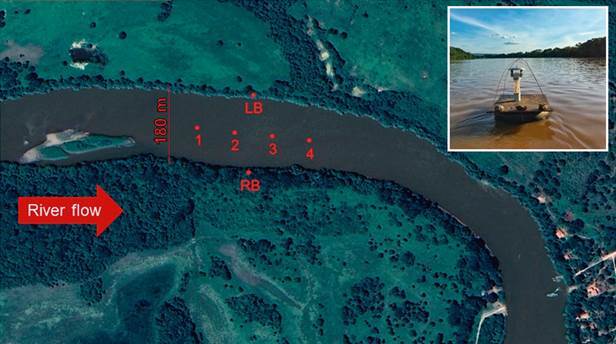
distribution of traps at the place of capture. (A) Trap installed in a sampling point in the central area of the Velhas’ River. (B) Place of distribution of the traps in the central region of Velhas’ River (1-4), on the right bank (RB) and left bank (LB), red arrow indicates the direction of river flow.

With the aid of a boat, four Hoover Penido (HP)-type light traps were placed approximately 80 metres apart in the centre of the river and one on each bank simultaneously (Fig. 5). To avoid false positive results, the traps were taken disassembled till the sample point at the centre of the river and set up before the sundown.

The position of traps and their distance from the banks of the Velhas’ River were measured using GPS (Garmin^®^). An anemometre and records from the Windy^®^ application were used to measure wind intensity and direction during collection periods and to determine possible influences, or not, on the flying capacity of sand flies.

*Storage validation of the phlebotomine marked* - A 50 mL Falcon^®^ tube containing a mixture of 20% glycerine in alcohol was used in each light trap as a container for recaptured sand flies. The possible dilution of the fluorescent powder on captured marked insects and the contamination of unmarked insects by marked insects was tested for six months using three containers with 20% glycerine in alcohol. One tube received 10 marked sand flies, another received eight marked and four unmarked sand flies, and the third received eight marked and 15 unmarked sand flies.

**Fig. 6:**
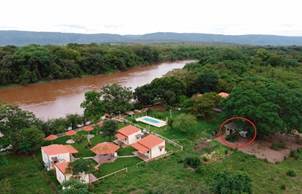
overview of “Pousada Tia Maria” on left bank of the Velhas’ River. Red circle indicates the hen house where sand flies were captured.

*Capture, marking, release and recapture* - The capacity of sand flies to carry out flights greater than 100 m in a continuously way was analysed at a location with high potential for the presence of sand flies and proximity to the riverbank. The selected location was Pousada Tia Maria, on the left riverbank, where a hen house, dogs and fruit trees were present, along with infrastructure for carrying out the investigation such as boats and dorms (Fig. 6).

**Fig. 7:**
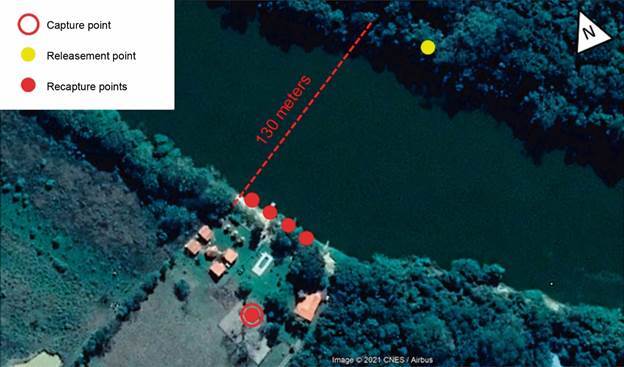
design of capture, mark, release and recapture analysis of sand fly flight capacity. Red circle represents the point of capture at the hen house. The yellow dot, located on right bank, 130 metres away from left riverbank, represents the point of release of marked sand flies. Red dots represent the locations of traps for attempted recapture of marked and released insects.

Collections were carried out from August 2020 to March 2021. Sand flies were captured at the hen house, on the left bank of the river, using three HP-type light traps along and active capture using Shannon trap per one night. The captured insects were stored alive in a breeding pot kept in a thermal box and fed with sugary solution. The insects were marked inside the breeding pots with fluorescent powder (Luminous Paint kit, Bioquip^®^), with the aid of a pear-shaped plastic sprayer. Different powder colours were used for the different months (red, yellow and blue). After marking, the insects were released on the right bank (release side) of the river and recapture traps were placed on left bank, inside the hen house and its’ surroundings (recapture side) (Fig. 7). Recaptures were performed for 48 h after release and 15 and 30 days after the first collection.

*Investigation of marked insects, preparation and identification of the insects collected during the experiments of capture-marking-release-recapture* - Captured insects were kept in the collecting tube with 20% glycerine alcohol mixture while insects collected during the flight capacity analysis were sacrificed in ether vapor and placed in a 50 mL Falcon tube containing 70º alcohol. After transport to the laboratory, the sand flies were analysed under a stereomicroscope using an ultraviolet light to observe the presence or absence of fluorescent marking. The flies were subsequently prepared, mounted in Berlese solution between a slide and cover slip. The flies were identified following the classification of Galati.^10^

## RESULTS

*Flight capacity* - The wind during the sampling period acted predominantly in a North-West direction with an average speed of 0.8 km/h (max.: 1.2 km/h and min.: 0.0 km/h) with gusts of 12.3 km/h throughout the day. January 2018 had the highest wind intensity (av 1.2 km/h), followed by July 2018 (av 0.7 km/h) and then May 2017 (av 0.6 km/h). The average maximum temperature during the sampling period was 26.6ºC while the average minimum temperature was 17ºC. The moon was in the waning crescent phase during the first sampling period and in the new phase (Table I) during the others. There was no rainfall during the sampling period.

**TABLE I:** Wind, temperature and Moon period during the sampling period for flight capacity analysis in Velhas’ River, Lassance, Minas Gerais, Brazil

Sampling period	Average Wind (gusts)	Wind direction	Average temperature (**º**C)	Moon
May 2017	0.6 k m/h (12 km/h)	Northwest	max: 27º min: 17º	◐ Waning crescent
January 2018	1.2 km/h (14 km/h)	Northwest	max: 27º min: 19º	● New
July 2018	0.7 km/h (11 km/h)	Northwest	max: 26º min: 15º	● New

Data collected by anemometer (wind-gage) and Windy: wind and weather forecast app^®^ data.

A total of 804 specimens were captured during the first attempt of the flight capacity analysis. The right bank had greater sampling success (65.7%) than the left bank (33.6%). The most abundant species were *Nyssomyia neivai* (63.43%) and *Ny. intermedia* (22.64%). Six specimens of three species were captured in the centre of the river, *Ny. neivai* (n = 4) followed by *Evandromyia lenti* and *Ny. intermedia* (n = 1 for both) (Table II). All specimens of *Ny. neivai* and *Ev. lenti* observed in centre of the river, were captured at point 1 in May 2017 while the specimen of *Ny. intermedia* was captured at point 4 in January 2018. The collection of July 2018 was not successful at sampling any flies in the centre of the river. Of all the sand flies captured in centre of the river, four (66.6%) were females and two (33.4%) were males.

**TABLE II:** Species of sand flies collected per sampling point during flight capacity analysis on the Velhas’ River, Lassance, Minas Gerais, Brazil

Species/Sampling point	1	2	3	4	RB	LB	TOTAL
*Brumptomyia* sp.	0	0	0	0	0	1	1
*Cortelezzii* complex	0	0	0	0	5	2	7
*Evandromyia evandroi*	0	0	0	0	3	0	3
*Evandromyia lenti*	1	0	0	0	6	4	11
*Evandromyia sallesi*	0	0	0	0	21	36	57
*Evandromyia teratodes*	0	0	0	0	1	0	1
*Evandromyia termitophila*	0	0	0	0	1	0	1
*Evandromyia walkeri*	0	0	0	0	25	2	27
*Lutzomyia longipalpis*	0	0	0	0	0	1	1
*Nyssomyia intermedia*	0	0	0	1	151	30	182
*Nyssomyia neivai*	4	0	0	0	314	192	510
*Pintomyia misionensis*	0	0	0	0	1	0	1
*Sciopemyia sordellii*	0	0	0	0	0	2	2
TOTAL	5	0	0	1	528	270	804

Attempts carried out in May 2017, January 2018 and July 2018. 1-4 represent the sample points among the centre of the river. RB: right bank; LB: left bank.

*Storage validation in 20% glycerine alcohol solution of the marked and unmarked sand flies* - The recapture methodology proved to be viable for the present study. No contamination was observed between marked and unmarked sand flies in recapture solution during the six-month period. There was also no dilution of the fluorescent powder in the recapture solution.

*Capture, mark, release and recapture* - Approximately 1,450 sand flies were captured by the Shannon and HP light traps, which were marked and released on the right bank during August 2020 (@ 500), December 2020 (@ 630), February 2021 (@ 200) and March 2021 (@ 120) (Table III).

**TABLE III:** Capture, release and recapture of sand flies on Velhas’ River, Lassance, Minas Gerais, Brazil, carried out in August 2020, December 2020, February 2021 and March 2021

	Sampling period			
	2020		2021	
	August	December	February	March
Number of sand flies marked and released (capture method)	≅ 500 (Shannon trap)	≅ 630 (Shannon and HP light trap)	≅ 200 (Shannon and HP light trap)	≅ 120 (HP light trap)
Number sand flies recaptured	-	-	-	-

HP: Hoover Penido.

The wind had a mild intensity during the recapture period, reaching an average of 0.9 km/h and acting in a North-West direction. A total of 1,029 sand flies were captured during the recapture attempts, but none were marked. The most abundant species during this study were *Ny. intermedia* (70.79%) and *Ny. neivai* (25.32%) and the most successful sampling site was the hen house and its surroundings (Table IV).

**TABLE IV:** Marked and non-marked sand flies captured during the recapture period on left bank of Velhas’ River, Lassance, Minas Gerais, Brazil, on the riverbank, hen house and its surroundings

	Riverbank		Hen house		Hen house surroundings		Marked sand flies	
	♀	♂	♀	♂	♀	♂		TOTAL
*Ny. intermedia*	24	14	182	332	94	81	-	727
*Ny. neivai*	34	84	11	48	24	59	-	260
*Ny. whitmani*	-	-	-	-	1	-	-	1
*Lu. longipalpis*	-	-	2	18	1	1	-	22
*Ev. sallesi*	-	1	-	-	-	1	-	2
*Mi. quinquefer*	-	-	1	-	-	-	-	1
*Mg. migonei*	-	-	1	-	-	-	-	1
*Ev. temithophila*	-	-	-	-	1	-	-	1
*Pa. limai*	-	-	2	1	-	-	-	3
*Cortelezzii* complex	-	-	3	-	4	-	-	7
*Brumptomyia* sp.	-	1	-	-	-	3	-	4
TOTAL	58	100	202	399	125	145	0	1029

## DISCUSSION

Studies that emphasise the flying ability of sand flies are scarce in the literature. Some mark-and-recapture analyses of dipterans in endemic areas have provided knowledge regarding the dispersion of these insects and the epidemiological implications of the distribution of medically important species, and therefore, of leishmaniasis.^11^ ^,^ ^12^ From this perspective, our in-situ analysis of non-marked sand fly flight, using an adapted support for light traps in an aquatic geographic barrier that prevents landing and rest by these insects during flight, is pioneering.

The applied methodology proved to be useful for measuring the capacity of sand flies to fly across a river as a geographical barrier. The light-trap support created here allows trapping sand flies over aquatic environments and can be used in wildlife studies on other flying insect groups, including those of medical importance. During one of our attempts, a high density of organic material was seen floating down the river, which became anchored to one of the supports causing the entire structure to sink. Therefore, as much as the support was useful, it can benefit from adaptations for similar future studies. We were, however, able reject the hypothesis of contamination of the capture tubes since the traps were disassembled, disconnected from the capture point and transported away at times of high solar incidence, which is unviable for the presence of sand flies.

Considering the estimated flight speed of 2.34 - 2.52 km/h for sand flies, as proposed by Killick-Kendrick et al.,^11^ the success of the six individuals captured in the centre of the Velhas’ River becomes plausible when considering that the minimum and maximum wind speed during the capture period was lower than these flight speeds. From the same perspective, winds with higher intensity than the flight speeds reported by Killick-Kendrick et al.^11^ could have a negative effect on sand fly flight by preventing, rather than assisting, their dispersion flight. The wind during the study acted predominantly in a North-West direction - from the right bank to the left bank. Thus, if there was positive wind interference, we believe that the sand flies captured in the centre of the river would have originated from the right bank. Still, we believe that, under the influence of strong winds, insects would not be able to be carried in the direction of the light traps and interrupt their “forced” flight to the point of being captured.

Colacicco-Mayhugh et al.^13^ also reported greater success sampling sand flies during periods of low winds when measuring interference by wind in the capture of insects. These authors also point out that high lunar illumination can negatively influence sampling of sand flies and generate a false indication of decreased activity of these insects, especially during periods of harvest moon. We did not observe any possible interference in the capture of sand flies in relation to lunar illumination since great abundances of various insects were found at the sampling points, although we did not capture during periods of harvest moon.

The area chosen for analysing sand fly flight by interception was very useful since it presented all the necessary characteristics already reported in planning the study, including easy access for installation and monitoring of traps. Saraiva et al.^14^ ^,^ ^15^ made sand fly captures on the banks of the Velhas’ River near our study area and found a local abundance and diversity of sand flies. Except for *Evandromyia teratodes*, all species found in the present study were also mentioned by Saraiva et al.^14^ Further corroborating these authors results, the species with the highest density in the same region were *Ny. neivai* and *Ny. intermedia*, which were also more abundant on right bank than on the left. The greater abundance of individuals for the right bank may be a reflection of a richer ecosystem or lesser human interference. A reversal of abundance was observed among *Ny. intermedia* and *Ny neivai* when observing the sampling site. It is noted that *Ny*. *neivai* is most abundant on the right bank. This fact may explain the dominance of *Ny. intermedia* species in the marking and release captures carried out on the left margin. Furthermore, the factors that influence the greater dominance of sand fly species in specific locations need to be better elucidated in a nearly future.

The recapture methodology of using a 50 mL falcon tube with a 20% glycerine alcohol mixture coupled to the HP light trap proved to be useful. This methodology causes less damage to captured specimens, maintains their morphological structures, and dispenses excessive handling.

Mark-recapture studies are quite laborious and require great attention and care in handling specimens to avoid damage and stress, thus increasing the chances of recapture. Perfilev^4^ discusses the flight range of sand flies by determining that they fly no more than 10 metres away based on studies of the release of marked insects in water bodies waiting to be captured on screen oiled paper at the nearest shore. However, this study does not discuss possible stress submitted to insects between the moments of capture - marking method - release. Still, it was not considered that sand flies could have greater attraction and greater stimulus to disperse using light traps for recapture. Here, success was not achieved in recapturing marked-and-released sand flies.

Another factor that may have been responsible for the lack of recaptures is the low number of insects that were marked-and-released each month. The release of a large number of marked individuals is necessary in such studies to even get a very low rate of recapture. Casanova et al.^12^ obtained a recapture rate of 16.2% for 6,502 marked-and-released sand flies, while Morrison et al.^16^ obtained a recapture rate of 5.5% for 3,747.

It is believed that many marked-and-released sand flies suffer from stress and die moments after release. Still, those that survive can find food sources on the spot and, thus, there is competition between local food sources and recapture traps, which reduces the chances of success. This corroborates with Galati et al.^17^ who discuss that some natural behaviour of adult sand flies such as displacement for colonisation, search for animal hosts or substrates for interbreeding and their quest for resting and breeding places may explain the low recapture rate in mark-release-recapture studies. Poché et al.^6^ demonstrated that *Phlebotomus argentipes* disperse among palm tree canopies, suggesting that some species of sand flies may use treetops as shelter and interbreeding and disperse through these microhabitats. These authors also demonstrate the behavioural difference of flights between somespecies of *Sergentomyia* genus flew towards the canopy in a hopping way while *P. argentipes* flew continuously towards the treetops. This shows that different species of sand flies may perform different forms of flight and specific studies should be conducted to observe the possible behavioural variables among sand fly species.

Other marking methods for insects have been studied and can reduce the stress of handling, such as spraying a mixture of sugary solution with coloured dye in vegetation where sand flies feed. Orshan et al.^18^ used this methodology and acquired some information about sand fly dispersion in a rural area. Nonetheless, this method would have been impracticable in other regions, such as Brazil, due to the great diversity of vegetation and the lack of knowledge about sand fly food preferences in relation to flora. Further studies are needed to clarify the relationship between flora and sand flies from the perspective of food options, resting sites, breeding sites and dispersion.

Casanova et al.^12^ reported a capacity for long-distance dispersion by *Ny. neivai* in rural areas and that the females cover greater distances than males. In our study, this species, together with *Ny. intermedia* and *Ev. lenti*, were captured at a distance of 90 metres from the riverbank, suggesting that these sand flies flew continually over this distance. It has been proposed that females can travel greater distances than males when searching for food sources, as reviewed by Ready,^9^ which may explain the higher proportion of females than males captured in the traps located in the centre of the river.

Previous studies have detected *Leishmania* DNA and/or the parasite itself in all species found in the present study except for *Ev. evandroi*, *Ev. teratodes* and *Ev. walkeri* ^19^ ^-^ ^33^ demonstrating the importance of these species to the epidemiology of leishmaniasis. Indeed, the most abundant species in the present study, *Ny. intermedia* and *Ny. neivai*, are considered medically important species because they present vectorial capacity for the parasite *Leishmania braziliensis*, which is known to cause cutaneous leishmaniasis in South and Central America^2^ ^,^ ^34^ and may be involved with the circulation of the parasite in wild and rural areas, such as the studied region.^15^

Finally, our study shows that sand flies have an ample flight capacity in their natural habitat, reaching a distance greater than previously reported in the literature. Such a finding expands knowledge about the dispersion of these insects and can help to infer the distribution of vector species and, therefore, the distribution of leishmaniasis in rural and/or urban areas. Furthermore, the present study emphasises the importance of conducting field studies in order to obtain a greater understanding of the ecology of pathogen-transmitting insects.

Data availability statement

The data that support the findings of this study are available from the corresponding author upon reasonable request.
